# Supplementary material for: Conditional chemoconnectomics (cCCTomics) as a strategy for efficient and conditional targeting of chemical transmission
Source: eLife. 2024 Apr 30;12:RP91927. doi: 10.7554/eLife.91927 (PMC11060718; doi:10.7554/eLife.91927)
Supplement: Figure 3—source data 3. [file elife-91927-fig3-data3.zip › Figure 3 source data3 part 1.zip!/Figure 3-Source data 3-part1/1063-13-CRISPResso_Report_8cz_fsdr.zip!/CRISPResso_on_1063_16/3a.Indel_size_distribution.pdf]

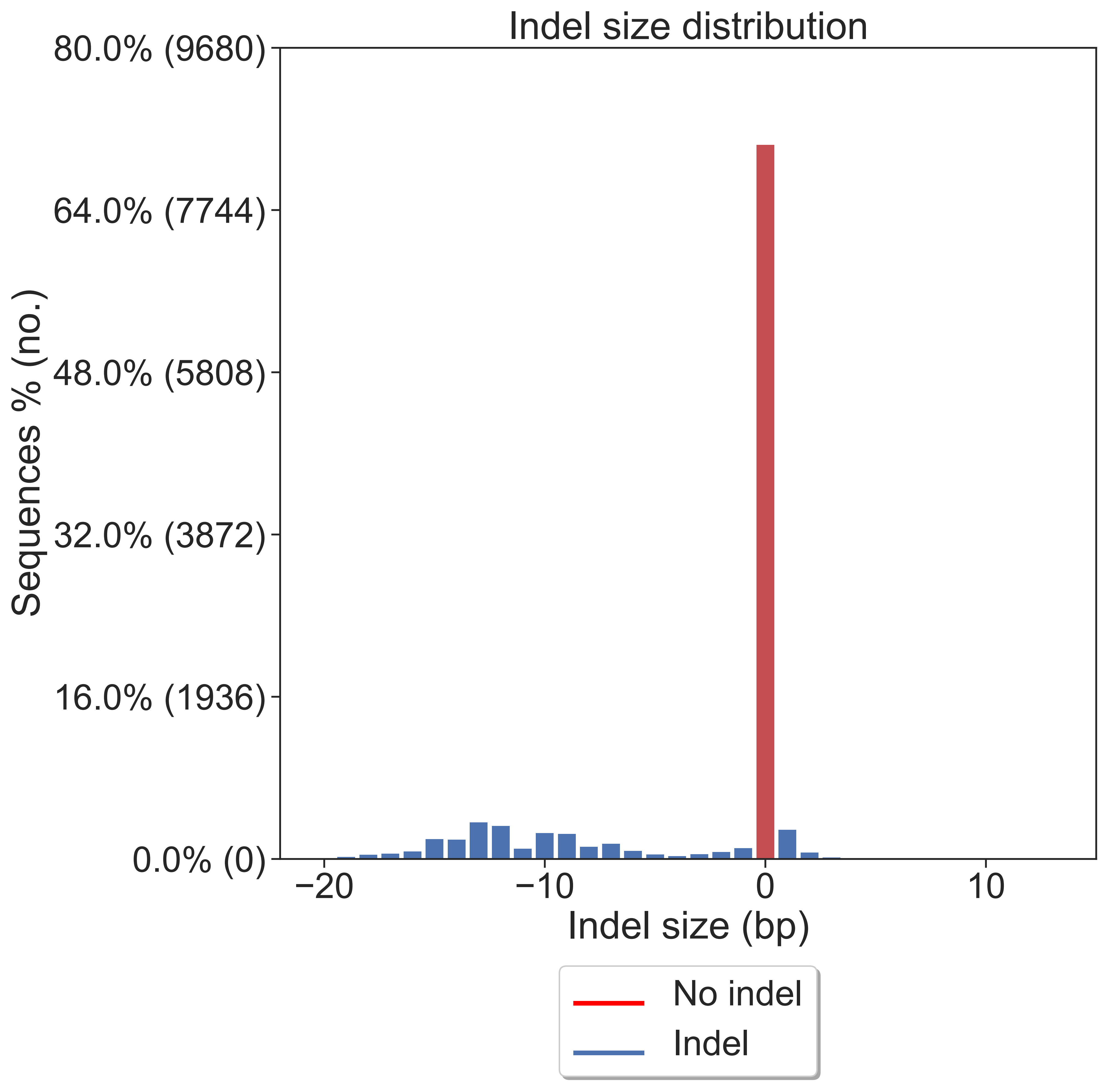

Indel size distribution
80.0% (9680)
64.0% (7744)
48.0% (5808)
32.0% (3872)
16.0% (1936)
0.0% (0)
Sequences % (no.)
−20
−10
0
10
Indel size (bp)
No indel
Indel
